# The prevalence of benign prostatic hyperplasia in mainland China: evidence from epidemiological surveys

**DOI:** 10.1038/srep13546

**Published:** 2015-08-26

**Authors:** Wenying Wang, Yuwen Guo, Daoxin Zhang, Ye Tian, Xiaonan Zhang

**Affiliations:** 1 Department of Urology, Beijing Friendship Hospital, Capital Medical University, Beijing, 100050, China; 2 College of Stomatology, Chongqing Medical University, Chongqing Key Laboratory of Oral Diseases and Biomedical Sciences

## Abstract

The epidemiological characteristics of benign prostate hyperplasia (BPH) in mainland China are not completely understood. We performed this meta-analysis to assess the prevalence of BPH from 1989 through 2014. A total of 14 articles and 19 datasets were included. The pooled overall prevalence of BPH among men aged 40 years and older was 36.6% [95% CI, 32.3–44.8]. The occurrence rate of BPH in the age groups 40–49 years, 50–59 years, 60–69 years, 70–79 years and 80 years and older was 2.9%, 29.0%, 44.7%, 58.1% and 69.2%, respectively. The pooled occurrence rate of BPH was 41.5% [95% CI, 34.5–48.4] in urban areas and 38.6% [95% CI, 22.7–54.6] in rural areas; this difference in prevalence was not statistically significant [OR, 1.51; 95% CI, 0.97–2.36]. BPH is highly prevalent in mainland China, and its prevalence increased with age. The trend in the prevalence of BPH in mainland China was not steady; the prevalence map based on a geographic information system (GIS) showed an unequal geographic distribution. High-quality surveys on BPH with a larger sample size are needed throughout mainland China to confirm these findings.

## Introduction

An increasing concern regarding illnesses related to the elderly population has been observed worldwide because of the unprecedented and pervasive trend of population aging in the 21^st^ century. Benign prostate hyperplasia (BPH) is the most common disease among aging males. It is reported that BPH occurs in 15% to 60% of men aged more than 40 years, and its prevalence increases markedly with age^1^,^2^. BPH is characterized by a benign overgrowth of prostatic tissue surrounding the urethra that ultimately constricts the urethral opening and is associated with lower urinary tract symptoms (LUTS), such as urgency, frequency, nocturia, incomplete bladder emptying, and weak urine stream^1^. Although it is not life-threatening, BPH is associated with serious morbidities, including an increased risk of falls, depression and diminished health-related quality of life, based on qualities such as sleep, psychological condition, activities in daily life, and sexual activities^3^,^4^,^5^. If BPH is left untreated, serious complications such as acute urinary retention (AUR), renal insufficiency and failure, urinary tract infection, and bladder stones can occur, requiring BPH-related surgical intervention^1^. This disease has high annual healthcare costs and places a considerable financial burden on the patients’ families and society^6^. Direct and indirect costs associated with BPH are approximately US $3.9 billion in the United States^7^ and £180 million in the UK^6^, and the substantial costs of diagnosis and treatment associated with BPH constitute an important public health issue in China. As people age, BPH has become an important global public health concern.

In recent decades, the aging population has increased rapidly in China due, in large part, to a decrease in mortality and an increase in life expectancy. According to the sixth national population census in 2010, 13.26% of the Chinese population was greater than 60 years old, which represents an increase of 2.93 percentage points from the fifth census in 2000 (http://www.stats.gov.cn/tjsj/tjgb/rkpcgb/qgrkpcgb/201104/t 20110428_30327.html). This percentage is estimated to reach 16.7% in 2020^8^. Population aging has been a challenge for healthcare systems in China. Because BPH is the most common disease in elderly males, understanding its prevalence has important implications for assessing the disease burden and planning national health care policy. The prevalence of BPH in China (6.6%) was first reported by Chang *et al.* in 1936 with 1900 mortality cases^9^. Another autopsy study in 1993 noted that the prevalence of BPH was 30.5%^10^, nearly five times higher than the level reported half a century before. The data in both reports were from inpatients; however, only studies based on the general population can reflect the actual situation of the disease. Since the 1980s, several epidemiological surveys on BPH have been conducted in different parts of China. The prevalence reports varied considerably, ranging from 20.57% in Pinghu^11^ to 66.95% in Tianjin^12^. The varying results may be related to differences among the studies in racial composition, age range, educational and economic levels, diagnostic criteria and sampling methods.

A national epidemiological survey of BPH has never been performed to date, and the data are limited and restricted to mainland China. The epidemiological characteristics of BPH remain incompletely understood. China occupies a vast territory and has the largest population in the world. Because of the high cost of diagnostic devices, it is difficult to conduct an epidemiological nationwide survey on BPH in the general population. Therefore, we conducted a systematic review of current evidence from regional population-based surveys on BPH to obtain a comprehensive picture of BPH in mainland China (excluding Hong Kong, Taiwan and Macao). The purpose of this study was to explore the overall prevalence of BPH in mainland China from 1989 through 2014 and to explore the discrepancy exhibited by age, survey time, urban vs. rural locations, and geographic distributions. The results may support the planning and implementation of public health policies and may identify future research priorities.

## Results

### Search results

**Figure 1:**
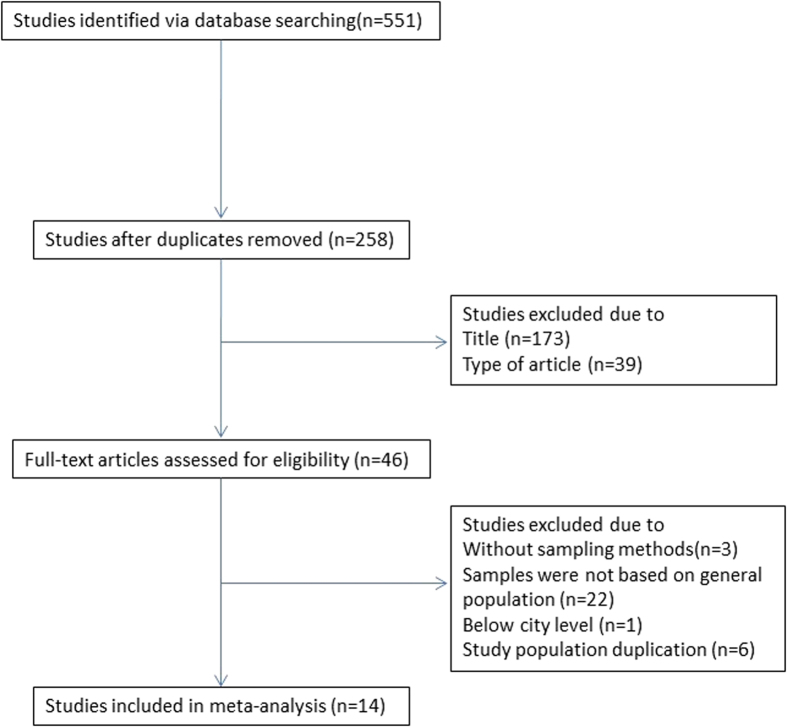
Flow chart of the article selection process for the prevalence of BPH in mainland China.

We identified 551 publications by searching PubMed (n = 83), CNKI (n = 151), the WanFang database (n = 212), the Chongqing VIP database (n = 48), and CBM (n = 57). We removed 293 duplicate records among the different databases. After examining the titles and abstracts, a total of 258 potentially eligible studies were selected for further evaluation. Finally, we identified a total of 14 studies in our meta-analysis that met the selection criteria^11^,^12^,^13^,^14^,^15^,^16^,^17^,^18^,^19^,^20^,^21^,^22^,^23^,^24^ (Fig. 1).

### Characteristics of included studies and assessment of risk bias

The characteristics of the fourteen studies and nineteen datasets included are shown in Table 1. All of the included studies were published from 1992 to 2014 in 13 provinces (Beijing, Gansu, Guangdong, Guangxi, Heilongjiang, Jilin, Liaoning, Shanghai, Shannxi, Sichuan, Tianjin, Xinjiang, and Zhejiang) in the following locations: Beijing, Pingliang, Guangzhou, Wuzhou, Haerbin, Baicheng, Shenyang, Shanghai, Xian, Chengdu, Tianjin, Urumchi, Xiaoshan, and Pinghu. Eleven publications reported using a specialist, urologist or trainee as the interviewer for the study. The participants in the included studies were aged at least 40 years. The selected publications used multi-stage, stratified, clustered, or randomized sampling methods, or some combination of these. Twelve studies reported a survey response rate of at least 90%. The diagnosis of BPH was primarily based on digital rectal examination (DRE), ultrasound examination or another clinical index, such as maximum flow rate (Qmax), prostate volume and International Prostate Symptom Score (IPSS). The prevalence of BPH in the selected fourteen studies ranged from 20.75% to 63.28%. The highest prevalence of BPH was found in Beijing, whereas the lowest prevalence of BPH was observed in Pinghu (Zhejiang province). Quality scores were used to assess the risk of bias in individual studies. Two studies had a quality score of 5, 3 studies had a score of 6, 2 studies had a score of 7, 6 studies had a score of 8, and 1 study had a score of 9; these results show that in general, the studies were of acceptable quality (Table 1).

### Overall prevalence of BPH

**Figure 2:**
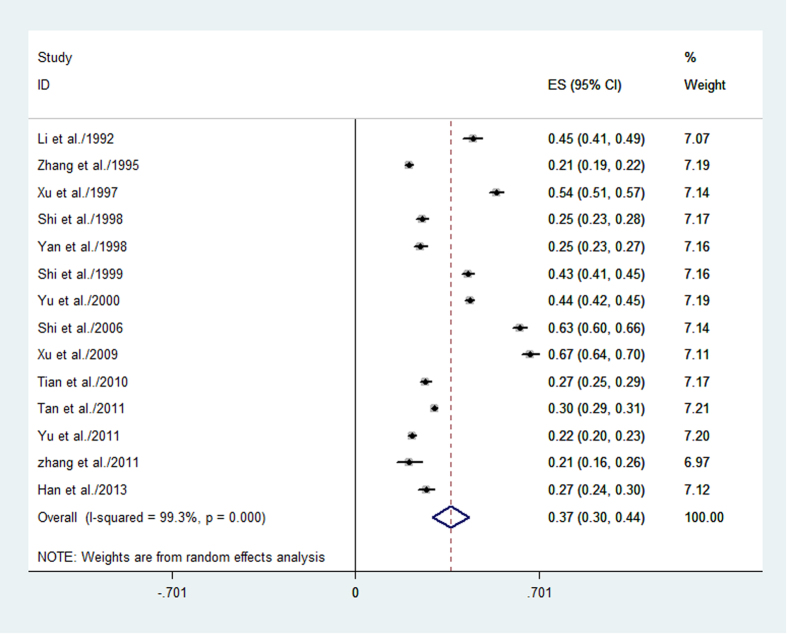
Forest plot for the overall estimate of the prevalence of BPH.

As shown in Table 2, a total of fourteen studies and nineteen datasets, which included a total sample size of 25040 and 8584 patients, evaluated the prevalence of BPH. The overall prevalence of BPH was 36.6% [95% CI, 32.3–44.8], and the forest plot for the overall estimates is shown in Fig. 2.

### Prevalence by location

**Figure 3:**
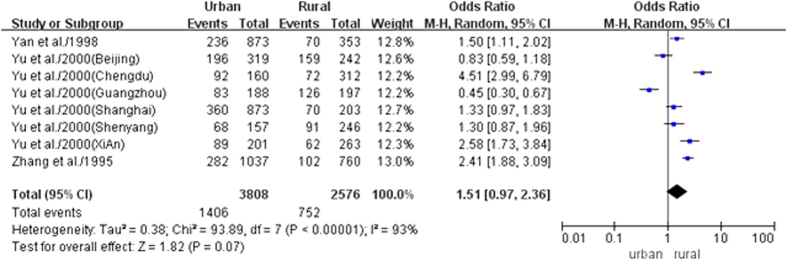
Comparison of the risk of BPH between urban and rural areas.

Fourteen datasets from nine studies provided the prevalence of BPH in urban areas, with a prevalence rate of 41.5% [95% CI, 34.5–48.4]. Nine datasets from four studies evaluated the prevalence of BPH in rural areas, with an estimate of 38.6% [95% CI, 22.7–54.6] (Table 2). There was no statistically significant difference in the prevalence of BPH between urban and rural areas [OR, 1.51; 95% CI, 0.97–2.36] (Fig. 3).

### Prevalence by age

**Figure 4:**
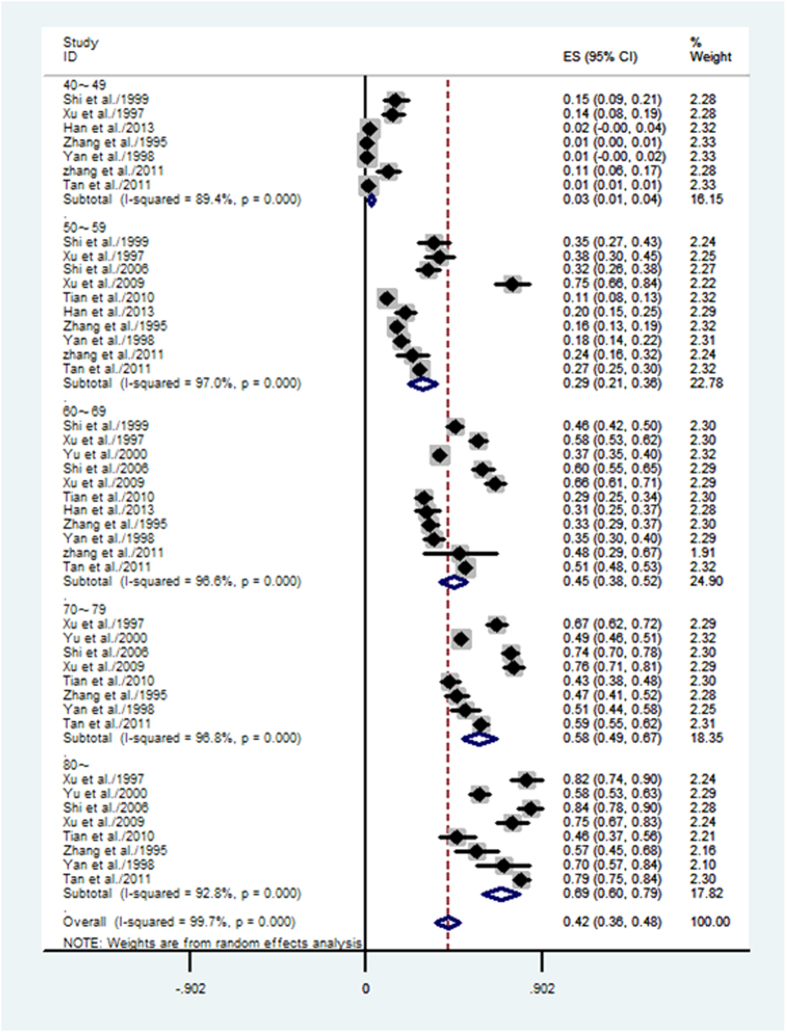
Prevalence of BPH by different age groups.

As shown in Table 2, the number of studies evaluating the age groups of 40–49 years, 50–59 years, 60–69 years, 70–79 years and 80 years and older were seven, ten, eleven, eight and eight, respectively. The prevalence of BPH in the age groups of 40–49 years, 50–59 years, 60–69 years, 70–79 years and 80 years and older were 2.9%, 29.0%, 44.7%, 58.1% and 69.2%, respectively (Fig. 4). The highest prevalence of BPH was 69.2% in the 80 years and older age group, whereas the lowest prevalence of BPH, 2.9%, was observed in the 40–49 years age group. Overall, the prevalence of BPH increased with advanced age.

### Subgroup analysis based on survey year, quality score of included studies, survey method and diagnostic criteria

As shown in Table 2, the prevalence of BPH in the survey year groups of 1981–1990, 1991–2000, 2001–2010 and 2010-present was 44.8%, 35.2%, 41.1% and 25.7%, respectively (Supplementary Fig. 1); the prevalence of BPH in the groups of 0–5 and 6–10 was 33.1% and 37.2%, respectively (Supplementary Fig. 2); the prevalence of BPH in the survey method groups of random, cluster random, stratified and multi-stage was 33.1%, 25.9%, 30.3% and 45.1%, respectively (Supplementary Fig. 3); the prevalence of BPH in the diagnostic criteria groups of A (prostate volume > 20 ml and medical history or questionnaire survey), B (prostate volume > 20 ml and Qmax < 15 ml/s or 10 ml/s and IPSS > 7) and C (criteria from academic conferences in China or the Guideline on BPH from the Chinese Medical Association, etc) was 23.7%, 39.5% and 45.2%, respectively (Supplementary Fig. 4).

### Trends in the prevalence of BPH

**Figure 5:**
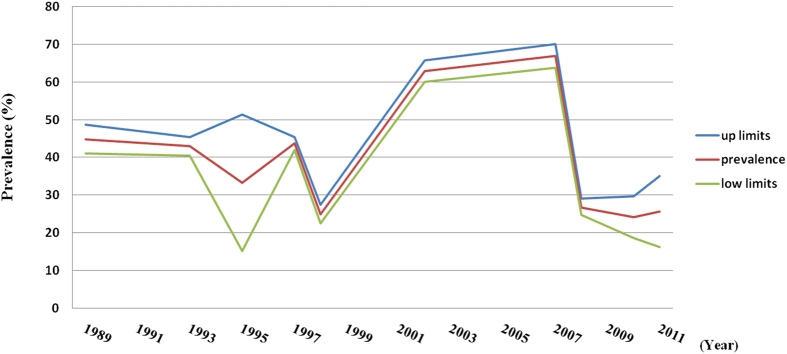
Pooled estimated prevalence of BPH in mainland China with corresponding 95% confidence intervals from different survey periods.

Figure 5 shows the trend in the overall estimated prevalence of BPH from 1989 through 2014 in mainland China. BPH was stable from 1989 to 1997, with the lowest prevalence (33.3%) in 1995 and the highest prevalence (44.8%) in 1989. In 1998, the prevalence of BPH decreased to 25.0%, which was also the lowest in the overall trend analysis. After four years, in 2002 the prevalence of BPH increased markedly to 62.9% and subsequently continued to increase steadily until 2007, when the prevalence was the highest (66.9%) in the overall trend analysis. However, the trend in the prevalence of BPH decreased in the period from 2008 to 2014. Generally, the trend in the prevalence of BPH in mainland China between 1989 and 2014 was not steady.

### BPH prevalence stratified by province in mainland China

**Figure 6:**
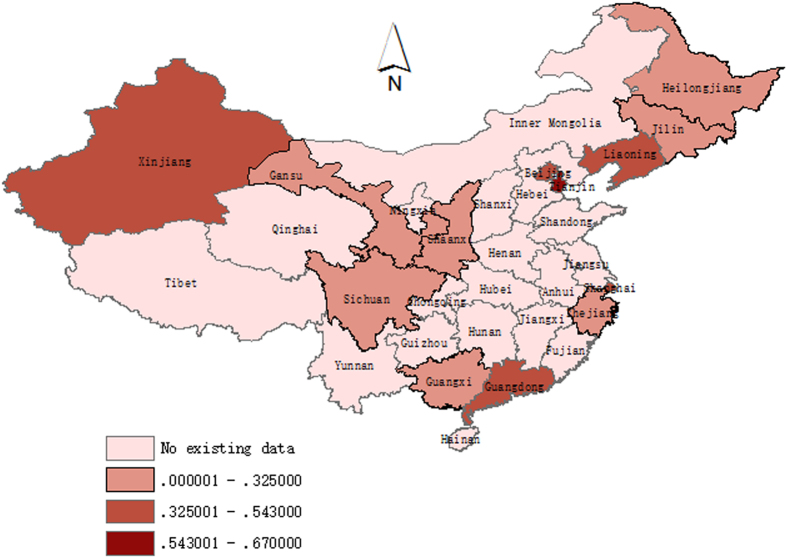
The provincial distribution of the prevalence of BPH on a map of mainland China (map was created by the ArcGIS software version 10 system).

Figure 6 shows a color-coded map illustrating the distribution of the prevalence of BPH in mainland China (data available in the following provinces: Beijing, Gansu, Guangdong, Guangxi, Heilongjiang, Jilin, Liaoning, Shanghai, Shannxi, Sichuan, Tianjin, Xinjiang, and Zhejiang). The prevalence of BPH in the provincial regions of mainland China ranged from 20.6% in the Zhejiang province to 67.0% in Tianjin. We created four distribution zones based on the prevalence of BPH. The first level represents no available data in the relevant regions and is pink on the map. The highest prevalence of BPH, observed in Tianjin and more than three times the prevalence in Zhejiang, belongs to the fourth level, shown on the map in the darkest red. Following the highest prevalence in Tianjin, the prevalence of BPH ranks the second highest in Guangdong (54.3%), then Shanghai (49.9%), Beijing (45.0%), Xinjiang (44.8%) and Liaoning (39.5%), which all belong to the third level. The second level distribution zone appears in light red on the map and includes Shannxi (32.5%), Guangxi (30.2%), Sichuan (29.9%), Gansu (27.2%), Heilongjiang (25.0%), Jilin (21.6%) and Zhejiang (20.6%). Overall, no particular concentration in the distribution of BPH prevalence was indicated on the map.

### Assessment of publication bias

We analyzed potential publication bias by generating funnel plots. The shape of the funnel plots was asymmetrical, which suggests the existence of publication bias. The existence of publication bias in this meta-analysis was also suggested by the results of an Egger’s test (p < 0.001).

## Discussion

To the best of our knowledge, this study is the first meta-analysis on the prevalence of clinical BPH in mainland China. Our results indicated that the pooled overall prevalence of BPH among men aged 40 years or older was 36.6% in mainland China during 1989 to 2014. The prevalence of BPH increased with age; the occurrence rate of BPH in the age groups of 40–49 years, 50–59 years, 60–69 years, 70–79 years and 80 years and older was 2.9%, 29.0%, 44.7%, 58.1% and 69.2%, respectively. No statistically significant difference was observed in the prevalence of BPH between urban and rural areas. The trend in the prevalence of BPH in mainland China was not steady. The prevalence map showed that the geographic distributions of BPH were unequal.

Strictly speaking, BPH is a histological diagnosis of hyperplastic glands that is only determined during autopsy studies^25^. In practice, BPH is typically diagnosed clinically on the basis of LUTS, and prostatic enlargement can be detected by manual rectal examination or trans-rectal ultrasonography. However, the occurrence rates of BPH reported in epidemiological studies were considerably different among different countries. Garraway *et al.*^26^ reported in a community-based study that the prevalence of BPH detected using transrectal ultrasonography was 25.3% for men aged 40–79 years in Scotland, whereas the prevalence of “prostatism” detected by spontaneous uroflowmetry was only 17% for men aged 50 years or more in a random-sampling study in Denmark^27^. In a multinational, community-based study using the IPSS questionnaire, the occurrence rates of BPH among men between the ages of 40 years and 79 years were 14%, 18%, 24%, 38%, and 56% in France, Scotland, Sweden, the US and Japan, respectively^28^,^29^. Another population-based study showed that the occurrence rates of BPH detected using the IPSS questionnaire and abdominal ultrasonography among Iranian men aged 40–49 years, 50–59 years, 60–69 years and ≥70 years were 1.2%, 18.45%, 26.8% and 36%, respectively (the overall prevalence was 23.8%)^30^. One explanation for the large variation in the reported prevalence is the lack of consensus on the definition and diagnostic criteria of clinical BPH in different investigations. Additionally, sample age, race, socio-economic status and sampling methods have a profound influence on the prevalence of BPH.

BPH typically begins in the fourth decade of life and is attributed to age-related dynamic changes in glandular tissue composition and cell proliferation^31^. A representative sample of Chinese men greater than 40 years of age was used in our study. In our systematic review, the prevalence of BPH among men aged 40 years or older in mainland China was high (36.6%). The higher prevalence in mainland China may be partially explained by racial differences. The cellular composition of the prostate may also play a role in the different occurrence rates of BPH. Lepo *et al.*^32^ reported that the mean prostate weights of Chinese men and Caucasian-American men were 53.4 g and 32.1 g, respectively, and the prostates of Chinese men contain significantly more glandular lumen and less smooth muscle and connective tissue. Yu *et al.*^33^ reported that the prostate tissue in Chinese men had higher glandular densities, whereas the prostate tissue samples of American men had a higher percentage of stroma. Several risk factors are associated with BPH, such as age, genetics, sex steroid hormones, and inflammation^4^. In the present meta-analysis, the prevalence of BPH continued to increase with age. With the population aging, the ratio of people greater than 60 years old is continuously increasing, which will cause a continuous increase in the of prevalence of BPH. In addition, the prevalence of BPH may vary due to differences caused by dietary structure, physical exercise, lifestyle choices, etc^4^,^34^. Increased physical exercise and lower levels of sedentary time have been robustly linked with a decreased risk of clinical BPH^34^,^35^. Some conventional Chinese behaviors, such as diets low in fruit and whole grain and physical inactivity, are potential reasons for the high prevalence of BPH^36^.

Previously, several studies reported that men in urban areas had a higher risk for BPH than those in rural areas^37^,^38^. However, with the rapid economic development and extraordinary pace of urbanization in rural areas, the difference in BPH between urban and rural areas has narrowed over time, and the typically higher rate of BPH observed in urban areas has recently disappeared^39^. In our systematic review, no statistically significant difference was found in the prevalence of BPH between urban and rural areas (41.5% vs 38.6%). This change may be caused by several factors. First, one likely explanation may be that lifestyle factors, such as dietary structure, particularly the increased daily intake of total calories, fat and animal protein, and the decreased daily intake of vegetables and whole grain, have been proven to play a role in the development of BPH and have changed in rural areas^40^,^41^,^42^. Second, an increased awareness of and screening for chronic aging diseases may be another potential reason for the increased prevalence, particularly for people in rural areas^43^. Third, the Chinese government is launching a project to build a cooperative medicare system in rural areas that will cover 900 million farmers with medical financial assistance; this program may help people in rural areas more easily seek medical advice, which could increase the diagnosis of BPH^43^. Finally, we should acknowledge the possibility that the discrepancy still exists but is undetected because only three studies with eight datasets investigated samples from both urban and rural areas in the same region. Because of the limited information in this systematic review, additional studies on elucidating the potential differences in the prevalence of BPH in urban and rural areas are warranted.

Because there has been no national epidemiological survey on BPH, its geographic distribution in mainland China remains unclear. In our study, we used meta-analysis to combine the data from all of the regional surveys on BPH in mainland China from 1989 to 2014, and we constructed a prevalence map of BPH using GIS. To some extent, this map can help to present the geographic distributions of BPH in mainland China and to identify the areas that most require health services. This map only provides information for thirteen provincial regions, and we could not find an obvious trend in the geographic distribution of BPH. The combined occurrence rates of BPH varied considerably in different regions; this variability may be partly due to differences in factors such as economic development, dietary structure, people’s awareness, and screening. Notably, there are large areas without data on the map because seventeen provinces had no epidemiological surveys on BPH. This fact suggests that those local governments have not realized the importance of this disease and that additional high-quality surveys should be conducted in those provinces without data.

In this meta-analysis, little to no “new information” regarding treatment options and quality of life (QOL) was presented because no such information can be extracted from the included studies. The impact of LUTS on daily activity, sexual function and psychology can lead to a decline in QOL in BPH patients^44^. After reviewing the treatment options available for BPH patients, no fundamental differences between Chinese and white populations were found; the three main options to treat BPH are watchful waiting, medical therapy and surgical treatment. A combination of alpha1-blockers and 5-alpha-reductase inhibitors is recommended, and Chinese herbal medicine is also used. While new surgical therapies are constantly emerging and being developed, transurethral resection of the prostate (TURP) rightly remains the dominant mode of treatment in mainland China^45^,^46^.

Heterogeneity should be considered in a meta-analysis, particularly in a meta-analysis of epidemiological studies^47^. Because our study was only performed to report the pooled prevalence in the general population and in various subgroups, meta-regression was not performed. The following factors contributed to the heterogeneity in this study: 1) Large differences existed in the sample sizes and in the age ranges in the included studies. 2) The validity of the data is heavily reliant on the sensitivity and specificity of the screening methods and diagnostic criteria; standardization of these methods would significantly reduce observational bias^48^. BPH has many different definitions, and there is currently no universally accepted case definition for BPH. A case definition can be applied widely in population-based epidemiologic studies^4^,^49^; diagnostic criteria, including radiographically determined prostate enlargement, physician-diagnosed BPH and urinary symptoms, were all used in the studies included in this meta-analysis. Due to this limitation, we pooled subsets that used the same or similar diagnostic criteria and reported those results. 3) We attempted to mitigate the heterogeneity by performing subgroup analysis; however, the heterogeneity was still high within subgroups based on survey time, survey method, age, urban/rural factor, diagnostic criteria and quality score of individual studies. Additionally, there were several other factors that likely contributed to heterogeneity, including racial factors, red meat and vegetable intake, smoking, marriage, career, exercise and obesity. However, it is not possible to analyze the effects of these factors on the pooled prevalence because of insufficient data.

The risk of bias was assessed by adapting a tool used in a previous meta-analysis^50^. The quality scores of most of the included studies were greater than or equal to 6 (a total score of 10 represents the lowest risk of bias), which indicated that the individual studies had a relatively low risk of bias. This meta-analysis may be subject to publication bias given that the included studies were sourced from journal publications only. A limited number of hospitals and institutes in mainland China have sufficient resources to conduct epidemiologic surveys of BPH, therefore higher-grade institutes and hospitals in developed regions may publish their studies more frequently in Chinese and English journals, resulting in some amount of publication bias.

Several other limitations should also be considered in this study. First, because not all of the provinces have conducted epidemiological studies on BPH, we only obtained data from 13 provinces in China. This limitation could have an impact on the results of our study. Second, although all of the participants were more than 40 years of age, the age range varied considerably among studies. Therefore, five discrete age groups were considered for comparison in our study. Third, nine studies reported the prevalence of BPH in urban areas, whereas only four studies provided data in rural areas. However, the rural population is dominant in mainland China. Fourth, screening instruments, diagnostic tools and the criteria for BPH have changed over time. Finally, the study design and the experience of the investigators may also have an effect on estimating the prevalence of BPH. Because of these limitations, we should be careful in interpreting these results and prescribing direct policy recommendations from this meta-analysis alone. Nevertheless, our study was conducted at an appropriate time because sufficient data were available to generate reasonably precise estimates of the prevalence of BPH. More high-quality epidemiologic and QOL studies on BPH with standard diagnostic criteria, similar methodology and large sample size are warranted throughout mainland China in the future, preferably in a prospective setting.

In summary, the present systematic review explored the epidemiological characteristics of BPH among males aged 40 years and older in mainland China. The results showed that the overall prevalence of BPH was high and increased significantly after the age of 50 years. No statistically significant difference was found between urban and rural areas. This review only provides a narrow window for the epidemiological status of BPH in China, with current studies from population-based surveys. In our opinion, our findings do reveal an important current healthcare issue that warrants the attention of policymakers.

## Methods

### Literature search and selection criteria

Our meta-analysis was performed following the guidelines from the Preferred Reporting Items for Systematic reviews and Meta-Analysis (PRISMA) statement^51^ (Supplementary Table 1). PubMed, Embase, the Chinese National Knowledge Infrastructure database (CNKI), the WanFang database, the Chongqing VIP database and the Chinese Biological Medical Literature database (CBM) were searched using the following search terms: “benign prostatic hyperplasia,” “BPH,” “prevalence,” “epidemiology,” “survey” and “China” from the established date through December 2014. Relevant articles in the reference lists were identified to obtain additional published studies.

The studies included in our analysis met the following criteria: 1) cross-sectional or longitudinal studies that were performed in mainland China; studies from Hong Kong, Taiwan and Macao were not included because of cultural differences from mainland China; 2) studies that provided sufficient information concerning the prevalence of benign prostatic hyperplasia; 3) studies based on the general population rather than volunteers; and 4) studies published in English or Chinese. The exclusion criteria were as follows: 1) studies not for BPH; 2) review or other article type; 3) studies without sampling methods; 4) studies performed with regard to a particular occupation, population, age group or specific area or otherwise not based on the general population; 5) studies performed below the city level, and 6) study population duplication.

### Data extraction

Two investigators independently performed the data extraction according to the selection criteria. Disagreements were resolved by discussion between those investigators or by consultation with another author until a consensus was reached. The following data from each included study were extracted: first author, year published, survey date, interviewer, location, age range, sample methods, diagnostic criteria, response rate, total sample size, and total case size. When necessary, we contacted the authors of the published studies to request relevant information for our analysis.

### Quality assessment of the included studies

A tool that was adopted from the Reporting of Observational Studies in Epidemiology (STROBE)^52^ guideline and was used in previous meta-analyses, such as the meta-analysis on diabetes by Li *et al.*, was used to assess the risk of bias for the individual studies^50^ (Supplementary Table 2).

The risk of bias was assessed by scoring each bias type for each study (high risk = 0, moderate risk = 1, and low risk = 2), and the total score represented the summary assessment of bias risk. The assessment was performed by two authors independently, and a final decision was reached by consensus when there was a disagreement.

### Statistical analysis

We performed calculations of the pooled prevalence on all of the included studies using the STATA software v. 11.1 (Stata, College Station, TX, USA) and Review Manager (RevMan) version 5.1. The random-effects meta-analysis was selected. Statistical heterogeneity was assessed using the I^2^ statistic, which was interpreted as low (25%–50%), moderate (51%–75%) or high (>75%) levels of heterogeneity. Subgroup analyses based on location, age, survey time, survey method, diagnostic criteria and quality score of the included studies were conducted. Publication bias was analyzed by generating funnel plots and measured using an Egger’s test; we considered P ≤ 0.05 to be significant. For exploring the spatial distributions of BPH in mainland China from 1989 through 2014, the pooled prevalence of BPH in each province was entered into the ArcGIS software version 10 system to construct the map of prevalence.

## Additional Information

**How to cite this article**: Wang, W. *et al.* The prevalence of benign prostatic hyperplasia in mainland China: evidence from epidemiological surveys. *Sci. Rep.* **5**, 13546; doi: 10.1038/srep13546 (2015).

## Supplementary Material

Supplementary Dataset 1

## Figures and Tables

**Table 1:** Characteristics of the included studies and quality scores for assessing the risk of bias in the individual studies.

Author/Year	Interviewer	Location	Age	Methods	Diagnostic criteria	RR	Sample size	Case size	Prevalence (per 100); [95%CI ]	Quality score*
Li *et al.*/1992	NA	Urumchi (U/R)	≥60	stratified cluster random	Standard national criteria in China	NA	647	290	44.82 [40.99–48.65]	5
Zhang *et al.*/1995	NA	Pinghu (U/R)	≥40	random	Prostate volume>20 ml, and Qmax<15 ml/s	100.00%	2217	456	20.57 [18.89–22.25]	7
Xu *et al.*/1997	specialist	Shanghai (U)	≥40	random	Prostate volume>20 ml, IPSS and Qmax<15 ml/s	98.88%	1218	656	53.86 [51.06–56.66]	8
Shi *et al.*/1998	specialist	Chengdu (U)	≥60	stratified cluster random	Criteria from academic conferences in China	90.00%	1448	369	25.48 [23.24–27.73]	8
Yan *et al.*/1998	NA	Haerbin (U/R)	≥40	random	Prostate volume>20 ml, and medical history	100.00%	1226	306	24.96 [22.54–27.38]	7
Shi *et al.*/1999	urologist	Shanghai (U)	≥40	multi-stage stratified random	Prostate volume>20ml, Qmax<10 ml/s, and medical history	98.90%	1582	680	42.98 [40.54–45.42]	8
Yu *et al.*/2000	specialist	six cities (U/R)	≥60	multi-stage cluster random	Prostate volume>20 ml and medical history	91.00%	3361	1468	43.68 [42.00–45.35]	9
		1) Beijing (U/R)					561	355	63.28 [59.29–67.27]	
		2) Shanghai (U/R)					1076	430	39.96 [37.04–42.89]	
		3) Guangzhou (U/R)					385	209	54.29 [49.31–59.26]	
		4) Chengdu (U/R)					472	164	34.75 [30.45–39.04]	
		5) XiAn (U/R)					464	151	32.54 [28.28–36.81]	
		6) Shenyang (U/R)					403	159	39.45 [34.68–44.23]	
Shi *et al.*/2006	specialist	Shanghai (U)	≥50	multi-stage cluster random	Prostate volume>20 ml, IPSS>7 and Qmax<10 ml/s	94.60%	1136	714	62.85 [60.04–65.66]	8
Xu *et al.*/2009	specialist and trainee	Tianjin (R)	≥50	multi-stage cluster random	Guideline on diagnosis and treatment of BPH from CMA	90.40%	832	557	66.95 [63.75–70.14]	8
Tian *et al.*/2010	specialist	Beijing (U/R)	≥50	multi-stage cluster random	Prostate volume>20 ml, IPSS>7, Qmax<15 ml/s	99.30%	1644	441	26.82 [24.68–28.97]	8
Tan *et al.*/2011	specialist	Wuzhou (U)	≥40	cluster random	Prostate volume>20 ml and medical history	91.40%	5826	1761	30.23 [29.05–31.41]	6
Yu *et al.*/2011	trainee	Baicheng (U)	≥60	cluster random	Criteria from specialized academic conferences in China	NA	2826	610	21.59 [20.07–23.10]	5
Zhang *et al.*/2011	specialist	Xiaoshan (U/R)	≥40	stratified cluster random	Prostate volume>20 ml and questionnaire survey	98.84%	257	53	20.62 [15.68–25.57]	6
Han *et al.*/2013	trainee	Pingliang (U/R)	≥40	multi-stage cluster random	Prostate volume>20 ml, IPSS>7, Qmax<15 ml/s	98.10%	820	223	27.2 [24.15–30.24]	6

NA: not available; Qmax: maximum flow rate; U: urban; R: rural; CMA: Chinese Medical Association; RR: response rate; IPPS: International Prostate Symptom Score; BPH: Benign Prostatic Hyperplasia.

^*^A total score of 0 represents the highest risk of bias and 10 represents the lowest risk of bias.

**Table 2:** Prevalence of benign prostatic hyperplasia in mainland China and subgroup analysis.

Variable		Number of surveys	Sample size	BPH cases	Prevalence (per 100) [95%CI ]	*I*^*2*^ (%)
Overall prevalence		14	25040	8584	36.6 [29.6–43.6]	99.3
Location	Urban	9	17844	6196	41.5 [34.5–48.4]	98.9
	Rural	4	3408	1309	38.6 [22.7–54.6]	99.2
Age	40–49	7	3596	84	2.9 [1.4–4.4]	89.4
	50–59	10	4093	961	29.0 [21.5–36.5]	97.0
	60–69	11	6166	2684	44.7 [37.8–51.6]	96.6
	70–79	8	4044	2274	58.1 [49.5–66.7]	96.8
	80–	8	1268	879	69.2 [59.8–78.6]	92.8
Survey Year	1981–1990	1	647	290	44.8 [41.0–48.7]	NA
	1991–2000	6	11052	3935	35.2 [24.8–45.7]	99.3
	2001–2010	5	7258	2545	41.1 [23.2–58.9]	99.6
	2011–present	2	6083	1814	25.7 [16.3–35.1]	92.7
Quality Score	0–5	2	900	3473	33.1 [10.4–55.9]	99.2
	6–10	12	7684	21567	37.2 [29.4–44.9]	99.3
Survey method	Random	3	4661	1418	33.1 [14.4–51.8]	99.5
	Cluster random	2	8652	2371	25.9 [17.5–34.4]	98.7
	Stratified	3	2352	712	30.3 [16.9–43.8]	97.7
	Multi–stage	6	9375	4083	45.1 [33.1–57.0]	99.3
Diagnostic criteria	A	4	5129	1192	23.7 [20.8–26.6]	78.6
	B	6	13623	4708	39.5 [28.2–50.8]	99.5
	C	4	6288	2684	45.2 [29.8–60.7]	99.3

BPH: benign prostatic hyperplasia; NA: not available; Diagnostic criteria A: Prostate volume > 20 ml and medical history or questionnaire survey.

B: Prostate volume > 20 ml and Qmax < 15 ml/s or10 ml/s and IPSS > 7.

C: Other criteria (criteria from academic conferences in China or the Guideline on diagnosis and BPH from the Chinese Medical Association).

## References

[b1] Wei J. T., Calhoun E. & Jacobsen S. J. Urologic diseases in America project: benign prostatic hyperplasia. J Urol 173, 1256–61 (2005). 15758764 10.1097/01.ju.0000155709.37840.fe

[b2] Parsons J. K., Bergstrom J., Silberstein J. & Barrett-Connor E. Prevalence and characteristics of lower urinary tract symptoms in men aged > or =80 years. Urology 72, 318–21 (2008). 18554695 10.1016/j.urology.2008.03.057 PMC2597492

[b3] Calais Da Silva F. *et al.* Relative importance of sexuality and quality of life in patients with prostatic symptoms. Results of an international study. Eur Urol 31, 272–80 (1997). 9129915 10.1159/000474467

[b4] Parsons J. K. Benign Prostatic Hyperplasia and Male Lower Urinary Tract Symptoms: Epidemiology and Risk Factors. Curr Bladder Dysfunct Rep 5, 212–218 (2010). 21475707 10.1007/s11884-010-0067-2 PMC3061630

[b5] Parsons J. K. *et al.* Lower urinary tract symptoms increase the risk of falls in older men. BJU Int 104, 63–8 (2009). 19154508 10.1111/j.1464-410X.2008.08317.x PMC3031126

[b6] Speakman M., Kirby R., Doyle S. & Ioannou C. Burden of male lower urinary tract symptoms (LUTS) suggestive of benign prostatic hyperplasia (BPH) - focus on the UK. BJU Int 115, 508–19 (2015). 24656222 10.1111/bju.12745

[b7] Saigal C. S. & Joyce G. Economic costs of benign prostatic hyperplasia in the private sector. J Urol 173, 1309–13 (2005). 15758787 10.1097/01.ju.0000152318.79184.6f

[b8] Pan J. G., Jiang C., Luo R. & Zhou X. Association of metabolic syndrome and benign prostatic hyperplasia in Chinese patients of different age decades. Urol Int 93, 10–6 (2014). 24246728 10.1159/000354026

[b9] Chang H. L. & Char G. Y. Benign hypertrophy of prostate. Chin Med J 50, 1707–1722 (1936).

[b10] Gu F. L., Xia T. L. & Kong X. T. Preliminary study of the frequency of benign prostatic hyperplasia and prostatic cancer in China. Urology 44, 688–91 (1994). 7526525 10.1016/s0090-4295(94)80207-6

[b11] Zhang D. Regional survey on the incidence of benign prostatic hyperplasia J Clin Urol 10, 364–365 (1995).

[b12] Xu Y. *et al.* Prevalence of benign prostate hyperplasia and its relative factors in rural areas of Tianjin in 2008. Chin J Urol 30, 761–764 (2009).

[b13] Han X. F., Ren J. L., Hu L. M., Chen F. R. & Xu K. X. [Prevalence of benign prostatic hyperplasia in Pingliang, Gansu: investigation and clinical analysis]. National J Androl 19, 324–7 (2013). 23678711

[b14] Zhang J., Xu H., Tang F., Zhang F. & Yao K. Investigation into the State of reproductive heaIth of adult males in XiaoShan District of Hangzhou. Chin J Adrol 25, 45–48 (2011).

[b15] Yu T., Li Z. & Liu B. Investigation and analysis of health status of elderly residents in a district. Guide of China Medicine 9, 269–270 (2011).

[b16] Tan R. *et al.* Investigation of benign prostatic hyperplasia among people over the age of 40 in Wuzhou. China Modern Medcine 18, 194–196 (2011).

[b17] Tian Y., Shao Q. & Song J. Prevalence of benign prostatic hyperplasia in BeiJing: a multicenter community-based cross-sectional survery. Chin J Urol 31, 194–198 (2010).

[b18] Shi Q. *et al.* Epidemiological survey of BPH in men over 50 years old in Pu Dong new area. Chin J Androl 20, 36–38 (2006).

[b19] Yu P. *et al.* [Prevalence of prostatic hyperplasia and its relative factors in six cities of China in 1997]. Zhonghua Liu Xing Bing Xue Za Zhi 21, 276–9 (2000). 11860799

[b20] Shi R., Wang Y. & Leng J. The Study of Epidemiology of Benign Prostatic Hyperplasia in Chinese Men in Shanghai Urban Area. J Shanghai Second Medical University 19, 270–272 (1999).

[b21] Yan Q., Zhang J. & Peng C. A regional survey on the incidence of benign prostatic hyperplasia Chinese primary health care 12, 41 (1998).

[b22] Shi Z., Zhang S. & Yang Y. [Study on health status of 3,333 old people in Chengdu City, Sichuan Province]. Zhonghua Liu Xing Bing Xue Za Zhi 19, 15–7 (1998). 10322699

[b23] Xu W. & Wang G. Survey of risk factors for benign prostatic hyperplasia in urban area of Shanghai. Shanghai J PREV MED 9, 107–110 (1997).

[b24] Li J., Yang F., Wang Y., Wang B. & Xu Q. Investigation of health status of 913 different ethnic elderly in Urumqi, Xinjiang. Chin J Gerontol 12, 72–73 (1992).

[b25] Lepor H. Pathophysiology, epidemiology, and natural history of benign prostatic hyperplasia. Rev Urol 6 Suppl 9, S3–S10 (2004). PMC1472917 16985922

[b26] Garraway W. M., Collins G. N. & Lee R. J. High prevalence of benign prostatic hypertrophy in the community. Lancet 338, 469–71 (1991). 1714529 10.1016/0140-6736(91)90543-x

[b27] Jensen K. M., Jorgensen J. B., Mogensen P. & Bille-Brahe N. E. Some clinical aspects of uroflowmetry in elderly males. A population survey. Scand J Urol Nephrol 20, 93–9 (1986). 2428099 10.3109/00365598609040555

[b28] Sagnier P. P. *et al.* International comparison of the community prevalence of symptoms of prostatism in four countries. Eur Urol 29, 15–20 (1996). 8821684 10.1159/000473711

[b29] Engstrom G., Walker-Engstrom M. L., Loof L. & Leppert J. Prevalence of three lower urinary tract symptoms in men-a population-based study. Fam Pract 20, 7–10 (2003). 12509363 10.1093/fampra/20.1.7

[b30] Safarinejad M. R. Prevalence of benign prostatic hyperplasia in a population-based study in Iranian men 40 years old or older. Int Urol Nephrol 40, 921–31 (2008). 18246438 10.1007/s11255-008-9338-7

[b31] Xia S. J., Xu X. X., Teng J. B., Xu C. X. & Tang X. D. Characteristic pattern of human prostatic growth with age. Asian J Androl 4, 269–271 (2002). 12508127

[b32] Lepor H., Shapiro E., Wang B. & Liang Y. C. Comparison of the cellular composition of benign prostatic hyperplasia in Chinese and Caucasian-American men. Urology 47, 38–42 (1996). 8560660 10.1016/s0090-4295(99)80379-3

[b33] Yu E. Y. *et al.* Histologic differences in benign prostate hyperplasia between Chinese and American men. Prostate 31, 175–9 (1997). 9167769 10.1002/(sici)1097-0045(19970515)31:3<175::aid-pros5>3.0.co;2-k

[b34] Lee H. W. *et al.* The Study About Physical Activity for Subjects With Prevention of Benign Prostate Hyperplasia. International Neurourology Journal 18, 155–162 (2014). 25279244 10.5213/inj.2014.18.3.155 PMC4180167

[b35] Parsons J. K. & Kashefi C. Physical activity, benign prostatic hyperplasia, and lower urinary tract symptoms. Eur Urol 53, 1228–35 (2008). 18358592 10.1016/j.eururo.2008.02.019

[b36] Yang G. *et al.* Rapid health transition in China, 1990-2010: findings from the Global Burden of Disease Study 2010. Lancet 381, 1987–2015 (2013). 23746901 10.1016/S0140-6736(13)61097-1 PMC7159289

[b37] Goh H. J., Kim S. A., Nam J. W., Choi B. Y. & Moon H. S. Community-based research on the benign prostatic hyperplasia prevalence rate in Korean rural area. Korean J Urol 56, 68–75 (2015). 25598939 10.4111/kju.2015.56.1.68 PMC4294858

[b38] Shan G., Deng F., Wang X. & Gu F. The urban and rural difference in elderly prostate volume growth. Chin J Urol 18, 134–136 (1997).

[b39] Wang J. An Epidemiological Study on Common Diseases of Elderly in Urban and Rural Areas of Chengdu. Chin J Prevent Contro 7, 267–269 (1999).

[b40] Gu F. Changes in the prevalence of benign prostatic hyperplasia in China. Chin Med J (Engl) 110, 163–6 (1997). 9594331

[b41] Parsons J. K. Lifestyle factors, benign prostatic hyperplasia, and lower urinary tract symptoms. Curr Opin Urol 21, 1–4 (2011). 21045705 10.1097/MOU.0b013e32834100c9

[b42] Raheem O. A. & Parsons J. K. Associations of obesity, physical activity and diet with benign prostatic hyperplasia and lower urinary tract symptoms. Curr Opin Urol 24, 10–4 (2014). 24247174 10.1097/MOU.0000000000000004

[b43] Wang Z. *et al.* Trends in prevalence, awareness, treatment and control of hypertension in the middle-aged population of China, 1992-1998. Hypertens Res 27, 703–9 (2004). 15785004 10.1291/hypres.27.703

[b44] Shao Q., Song J., Guo Y., Lv W. & Du L. The evaluation of quality of life in men with symptomatic benign prostatic hyperplasia. Chin J Urol 27, 418–420 (2006).

[b45] Xia S. J., Cui D. & Jiang Q. An overview of prostate diseases and their characteristics specific to Asian men. Asian J Androl 14, 458–64 (2012). 22306914 10.1038/aja.2010.137 PMC3720159

[b46] Ma C. H. *et al.* Efficacy and safety of Chinese herbal medicine for benign prostatic hyperplasia: systematic review of randomized controlled trials. Asian J Androl 15, 471–82 (2013). 23728585 10.1038/aja.2012.173 PMC3739225

[b47] DerSimonian R. & Laird N. Meta-analysis in clinical trials. Control Clin Trials 7, 177–88 (1986). 3802833 10.1016/0197-2456(86)90046-2

[b48] Chan K. Y. *et al.* Epidemiology of Alzheimer’s disease and other forms of dementia in China, 1990-2010: a systematic review and analysis. Lancet 381, 2016–23 (2013). 23746902 10.1016/S0140-6736(13)60221-4

[b49] Chokkalingam A. P. *et al.* Prevalence of BPH and lower urinary tract symptoms in West Africans. Prostate Cancer Prostatic Dis 15, 170–6 (2012). 21912428 10.1038/pcan.2011.43 PMC6314026

[b50] Li H. *et al.* Diabetes prevalence and determinants in adults in China mainland from 2000 to 2010: a systematic review. Diabetes Res Clin Pract 98, 226–35 (2012). 22658670 10.1016/j.diabres.2012.05.010

[b51] Liberati A. *et al.* The PRISMA statement for reporting systematic reviews and meta-analyses of studies that evaluate healthcare interventions: explanation and elaboration. BMJ 339, b2700 (2009). 19622552 10.1136/bmj.b2700 PMC2714672

[b52] von Elm E. *et al.* The Strengthening the Reporting of Observational Studies in Epidemiology (STROBE) statement: guidelines for reporting observational studies. Lancet 370, 1453–7 (2007). 18064739 10.1016/S0140-6736(07)61602-X

